# Chloroquine Triggers Cell Death and Inhibits PARPs in Cell Models of Aggressive Hepatoblastoma

**DOI:** 10.3389/fonc.2020.01138

**Published:** 2020-07-17

**Authors:** Katja Eloranta, Stefano Cairo, Emmi Liljeström, Tea Soini, Antti Kyrönlahti, Jean-Gabriel Judde, David B. Wilson, Markku Heikinheimo, Marjut Pihlajoki

**Affiliations:** ^1^Pediatric Research Center, Children's Hospital, Helsinki University Hospital, University of Helsinki, Helsinki, Finland; ^2^Xentech, Evry, France; ^3^Department of Medicine, Center for Infectious Medicine, Karolinska Institutet, Stockholm, Sweden; ^4^Department of Pediatrics, Washington University School of Medicine, St. Louis Children's Hospital, St. Louis, MO, United States; ^5^Department of Developmental Biology, Washington University School of Medicine, St. Louis, MO, United States

**Keywords:** liver cancer, hepatoblastoma, pediatric oncology, poly(ADP)-ribose polymerase, metabolomics, aspartate, NAD^+^

## Abstract

**Background:** Hepatoblastoma (HB) is the most common pediatric liver malignancy. Despite advances in chemotherapeutic regimens and surgical techniques, the survival of patients with advanced HB remains poor, underscoring the need for new therapeutic approaches. Chloroquine (CQ), a drug used to treat malaria and rheumatologic diseases, has been shown to inhibit the growth and survival of various cancer types. We examined the antineoplastic activity of CQ in cell models of aggressive HB.

**Methods:** Seven human HB cell models, all derived from chemoresistant tumors, were cultured as spheroids in the presence of relevant concentrations of CQ. Morphology, viability, and induction of apoptosis were assessed after 48 and 96 h of CQ treatment. Metabolomic analysis and RT-qPCR based Death Pathway Finder array were used to elucidate the molecular mechanisms underlying the CQ effect in a 2-dimensional cell culture format. Quantitative western blotting was performed to validate findings at the protein level.

**Results:** CQ had a significant dose and time dependent effect on HB cell viability both in spheroids and in 2-dimensional cell cultures. Following CQ treatment HB spheroids exhibited increased caspase 3/7 activity indicating the induction of apoptotic cell death. Metabolomic profiling demonstrated significant decreases in the concentrations of NAD^+^ and aspartate in CQ treated cells. In further investigations, oxidation of NAD^+^ decreased as consequence of CQ treatment and NAD^+^/NADH balance shifted toward NADH. Aspartate supplementation rescued cells from CQ induced cell death. Additionally, downregulated expression of PARP1 and PARP2 was observed.

**Conclusions:** CQ treatment inhibits cell survival in cell models of aggressive HB, presumably by perturbing NAD^+^ levels, impairing aspartate bioavailability, and inhibiting PARP expression. CQ thus holds potential as a new agent in the management of HB.

## Introduction

Hepatoblastoma (HB) is a malignant liver neoplasm that usually occurs in children younger than 4-years (1). It is the most common primary hepatic malignancy in the pediatric population with an annual incidence of 1.2–1.5/1,000,000 (2). Risk factors for HB include low birth weight, pre-maturity, and certain congenital disorders such as Beckwith-Wiedemann syndrome and familial adenomatous polyposis (FAP) (3–5). Complete tumor resection is paramount for optimal outcome, but majority of HBs are not surgically treatable at the time of diagnosis (6–8). Platinum-derivatives, cisplatin, and carboplatin, combined with doxorubicin form the backbone of neoadjuvant and adjuvant chemotherapy in HB treatment (9). Nonetheless, severe adverse effects limit the use of these agents, and chemoresistance is a hallmark of aggressive HB (10–12). Thus, new and less toxic therapeutic modalities would be desirable to further improve the results of HB management.

Chloroquine (CQ), a drug discovered in 1934, has been used broadly in the treatment of malaria, rheumatoid arthritis, and systemic lupus erythematosus (13–16). CQ also has documented antineoplastic activity in various solid cancer types including hepatocellular carcinoma, non-small cell lung cancer, glioblastoma multiforme, bladder cancer, pancreatic adenocarcinoma, and colon cancer (17–23). In addition to its efficacy as monotherapy, CQ has shown potential to sensitize cancer cells to conventional therapy after primary treatment has failed (24–28). Currently, there are 21 clinical trials evaluating efficacy and safety of CQ in oncology practice (Supplementary Table 1).

Multiple mechanisms underlying the tumor suppressive activity of CQ have been described. Autophagy is a process linked to metastasis and activation of chemoresistance in a context of aggressive cancer, and its inhibition is a widely recognized mechanism of CQ action (29–32). *In vitro* studies have shown that the transforming growth factor beta (TGF-β), hedgehog, and p53 signaling pathways are affected by CQ treatment (33–37). Other biological processes impacted by CQ administration include G2/M cell cycle arrest, increased apoptosis, altered inflammatory responses, and tumor vessel normalization (38–42).

Previous studies have shown that autophagy promotes survival of immortalized HB cells and tumor growth *in vivo*, suggesting that therapeutic interventions disturbing autophagic flux may hold potential in HB management (43, 44). In this study, we demonstrate the efficacy of CQ in 3-dimensional (3D) cell models of HB established from patients whose response to the first line treatments was suboptimal. Further, we shed new light on the molecular mechanisms of CQ action in HB cells.

## Materials and Methods

### Cell Cultures and Treatments

Immortalized human HB cell line HUH6 was purchased from Japanese Collection of Research Bioresources Cell Bank (Osaka, Japan) and maintained as described earlier (45). Recently established human HB cell lines HB-284, HB-282, HB-303, HB-243, HB-295, and HB-279 were obtained through collaboration with XenTech (Evry, France) and cultured in Advanced DMEM/F12 (Gibco, Waltham, MA, USA) supplemented with 8% fetal bovine serum, 2 μM L-glutamine, 100 units/ml penicillin, 100 μg/ml streptomycin sulfate, and 20 μM rock kinase inhibitor Y-27632 (SelleckChem, Houston, TX, USA). Cultures were maintained at 37°C in a humified incubator containing 5% CO_2_.

Cells were treated with CQ diphosphate (#ab142116; Abcam, Cambridge, MA, USA) dissolved in sterile water as a 10 mM stock solution. Further dilutions were prepared in adequate cell culture medium. Cell culture medium without CQ served as a control treatment. For all the experiments fresh culture medium with or without CQ was replaced daily.

### 3D Spheroid Cultures

Cells were seeded at density of 3,000 cells/well to low attachment CellCarrier spheroid ULA 96-well-plates (PerkinElmer, Waltham, MA, USA). After 72 h incubation, spheroids were established and treatment with CQ or control medium was initiated. Images were captured at treatment timepoints of 0, 48, and 96 h with Eclipse TS100 microscope supplemented with DS-Fi1 digital imaging system (Nikon, Tokyo, Japan).

### 3D Viability Measurements

Viability of spheroids was assessed with ATPlite™ 3D monitoring system (PerkinElmer) as described in the manufacturer's instructions at treatment timepoints of 48 and 96 h. Luminescence was measured with EnSpire Multimode Plate Reader (PerkinElmer).

### Apoptosis Assay

Caspase 3/7 Glo assay (Promega, Madison, WI, USA) was used to measure apoptosis in 3D cultures as instructed. Luminescence was recorded with EnSpire Multimode Plate Reader (PerkinElmer).

### Clonogenic Cell Survival Assay

HUH6, HB-284, and HB-243 cells were seeded at low density in 12-well-plates and cultured for 4 or 14 days after attachment in absence of CQ or with CQ in concentrations 1, 5, or 10 μM. Cells were fixed with 4% paraformaldehyde, permeabilized with 100% methanol, and subsequently stained with crystal violet. Images were captured with Bio-Rad ChemiDoc XRS+ Imaging System (Bio-Rad, Hercules, CA, USA). Colony number was determined with ColonyArea Plugin in ImageJ Software (46).

### Metabolomic Profiling

HUH6 cells were treated with control medium or 5 μM CQ for 96 h. Next, 1.5 × 10^6^ cells/sample were collected, and cell pellets were snap-frozen in liquid nitrogen for analysis. Acquity UPLC-MS/MS system and XEVO TQ-S Triple Quadrupole LC/MS (Waters Corporation, Milford, MA, USA) were used to analyze the samples. Normalized concentrations of 100 metabolites were analyzed with web-based software MetaboAnalyst 4.0 (http://www.metaboanalyst.ca) (47). Detailed information of sample processing, reagents, and full protocol are given elsewhere (48).

### NAD/NADH Assay

NAD/NADH ratio was measured with NAD/NADH assay kit purchased from Abcam. Briefly, HUH6 cells were cultured with or without 5 μM CQ for 96 h. Cells were lysed and processed following manufacturer's instructions as described (49). The reaction was allowed to develop for 1.5 h, and then absorbance was measured at 450 nm with Multiskan FC microplate reader (Thermo Fisher Scientific, Vantaa, Finland). NAD/NADH ratio was calculated using normalized concentrations by equation ([NADtotal—NADH])/[NADH].

### Aspartate Rescue Experiment

Cells were treated with 5 μM CQ or 5 μM CQ supplemented with 10 mM L-aspartic acid (Sigma Aldrich, St. Louis, MO, USA) for 96 h. The impact of 10 mM L-aspartic acid alone on growth was also monitored. Cell viability was assessed by ATPLite Luminescence assay system using Enspire Multimode Plate Reader (both from PerkinElmer).

### RNA Extraction and RT-qPCR

Total RNA was extracted from HUH6 cells treated with 5 μM CQ or control media for 96 h utilizing RNAeasy Mini Kit (QIAGEN, Valencia, CA, USA) as instructed. Reverse transcription was performed with Reverse transcriptase Core kit (Eurogentec, Seraing, Belgium). RT^2^ Profiler Cell Death Pathway Finder qPCR array (QIAGEN) was performed as described in the manufacturer's instructions. Geometric mean of *B2M, HPRT1*, and *GAPDH* expression served as a reference.

### Protein Extraction and Western Blotting

Proteins were extracted utilizing NucleoSpin RNA/Protein extraction kit (Macherey-Nagel, Düren, Germany). Ten micrograms of protein was separated by electrophoresis using Mini-Protean TGX Stain-Free Gels (Bio-Rad). Proteins were transferred onto polyvinyl fluoride membrane and non-specific binding was blocked with 5% non-fat milk in 0.1% Tris-buffered Tween saline buffer. Membranes were incubated with following primary antibodies at +4°C for overnight: anti-human PARP1 rabbit IgG in dilution 1:1,500 (#9532; Cell Signaling Technology, Danvers, MA, USA) and anti-human PARP2 rabbit IgG in dilution 1:1,000 (#NBP2-47337; Novus Biologicals, Littleton, CO, USA). Next, goat anti-rabbit IgG secondary antibody (#111-035-144 in dilution 1:10,000; Jackson ImmunoResearch, West Grove, PA, USA) incubation was performed at room temperature for 1 h. Protein bands were detected utilizing Enhanced Chemiluminescence detection kit (Amersham ECL reagent; GE Healthcare, Barrington, IL) and analyzed with Image Lab Software 6.0 (Bio-rad). Band intensity was normalized to total protein amount in each lane.

### Immunofluorescence Staining

HUH6 cells (200,000/well) were grown with or without 5 μM of CQ for 96 h on 2-well-chamber slides pre-coated with Matrigel (Corning, Corning, NY, USA). Subsequently, cells were fixed and permeabilized with ice-cold 100% methanol (5 min, room temperature). Unspecific binding was blocked with UltraVision Protein Block (Thermo Fisher Scientific). Next, cells were incubated with primary antibody at room temperature for 1 h (#9532 human anti-rabbit PARP1 at 1:800 dilution, Cell Signaling Technologies). Secondary antibody incubation was performed with goat anti-rabbit IgG (H+L) AlexaFluor 647 (1 h, room temperature) at 1:500 dilution (A32733, Invitrogen, Carlsbad, CA, USA). Images were captured with Zeiss Axio Imager M2 (objective: EC Plan Neofluar 40 × /0.75 Ph 2 M27) (Carl-Zeiss, Oberkochen, Germany).

### Statistical Analyses

For viability assays, apoptosis measurements, and RNA and protein quantifications statistical analyses were performed using JMP Software (JMP Pro; version 14.1.0, SAS Institute Inc.). All the experiments were carried out at least in triplicate. Statistical significance was assessed using Student's *t*-test or one-way ANOVA depending the experimental design. ^*^P < 0.05 was considered as a statistically significant and ^**^p < 0.01 as a statistically highly significant. Metabolomic profiling analyses were completed using MetaboAnalyst 4.0. software (47).

## Results

### HB Spheroids Treated With CQ Show Decreased ATP Availability and Increased Cell Death

**Figure 1:**
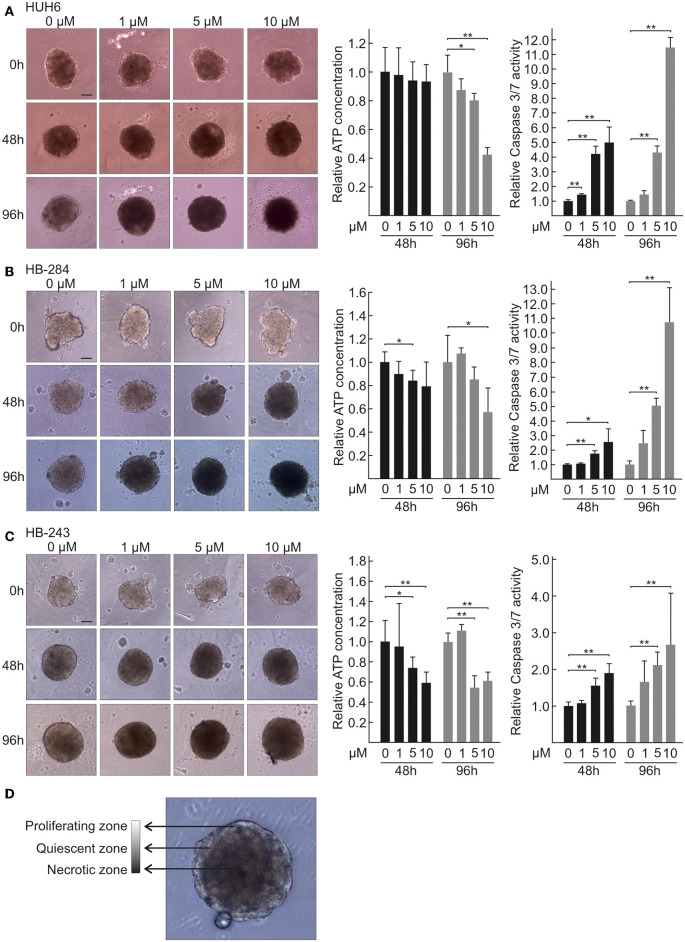
Morphology, viability, and caspase 3/7 activation of HB spheroids treated with CQ. Morphology of HUH6 **(A)**, HB-284 **(B)**, and HB-243 **(C)** derived spheroids treated with control medium (0 μM) or CQ at concentrations of 1, 5, and 10 μM (left panel). Relative ATP concentration (middle panel) and relative caspase 3/7 activation (right panel) in HUH6 **(A)**, HB-284 **(B)**, HB-243 **(C)** derived spheroids after 48 and 96 h CQ treatment. CQ concentrations; 1, 5, and 10 μM. *P < 0.05. **P < 0.01. Statistical significance was assessed with one-way ANOVA. Bar plots are presented as relative values of mean ± RSD (*N* = 3). Characteristics of proliferating, quiescent, and necrotic spheroid morphology **(D)**. Pictures were captured at initiation of treatment (0 h) and after 48 h and 96 h of CQ administration. Magnification 10×, scale bar = 10 μm.

Since traditional 2D cell cultures do not accurately represent the architecture and interactions of solid tumors, we assayed the effect of CQ treatment using 3D HB spheroid models. Six of the cell lines used in this study were established from aggressive HB tumors (Clinical details in Table 1) thus representing a situation of an unfavorable treatment outcome for first line therapy (50). The seventh cell line, HUH6, is a gold standard in HB research. Spheroids were treated with three different concentrations of CQ in an acceptable range (51). Spheroid morphology was monitored after 48 and 96 h of treatment by capturing brightfield images with inverted phase contrast microscope. CQ treatment triggered a time and dose-dependent increase in the necrotic non-viable zone and loss of proliferative edge in all 7 spheroid models studied (Figures 1A–C, Supplementary Figures 1A–D). Characteristics of proliferating, quiescent, and necrotic spheroid morphology are shown in Figure 1D. Next, the viability of HB spheroids was quantified by ATP measurements. After 48 h of CQ treatment a statistically significant decrease in viability was observed in two out of seven models (Figures 1B,C). By 96 h, six out of seven models showed a significant decrease in viability (Figures 1A–C, Supplementary Figures 1A,B,D). Since increased apoptotic cell death has been reported following CQ treatment (40, 52), we next measured activation of caspase 3/7 in HB cell spheroids. After 48 h, all 7 models showed a significant increase in apoptosis with 10 μM CQ compared to control (Figures 1A–C, Supplementary Figures 1A–D). An even more enhanced caspase 3/7 activation was detected in four out of seven cell models (Figures 1A–C, Supplementary Figure 1A) after 96 h, and three out of these four proceeded toward apoptotic cell death at a lower CQ concentration (5 μM).

**Table 1:** Clinical information of HB patients.

**Sample ID**	**HB-303**	**HB-243**	**HB-279**	**HB-282**	**HB-284**	**HB-295**
Age (months)	69	52	79	12	83	26
Type of sample	Primary	Intrahepatic relapse	Primary	Primary	Peritoneal metastasis at relapse	Primary
R, resection; LT, liver transplant	R	LT	LT	R	R	R
Sex	F	M	M	M	M	F
Vascular invasion Y/N	N	Y	Y	N	n/a	Y
S, solitary; M, multiple nodules	M	M	M	S	n/a	M
Metastasis Y/N	N	N	N	N	n/a	Y
Main histological component	Fetal	Embryonal	Embryonal + Macrotrabecular	Embryonal	Embryonal	Fetal
PRETEXT	II	n/a	IV	II	n/a	II
Chemotherapy protocol	Cisplatin 4 cycles	Carboplatin + Etoposide	SIOPEL-4	SIOPEL-3 + SIOPEL-6	Etoposide + Cisplatin	SIOPEL-4
AFP serum at diagnosis (ng/mL)	158,645	6,000	1,000,000	1,286,000	2,162	585,350
AFP serum post-chemoth. (ng/mL)	26,000	5,000	30,000	1,000,000	1,089	1,400

*PRETEXT, Pre-treatment extent of disease, radiological staging system for primary pediatric liver malignancies. AFP, alpha-fetoprotein. n/a, information is not available. Information adapted from (50)*.

### CQ Treatment Impairs Viability in HB 2D Cell Cultures

**Figure 2:**
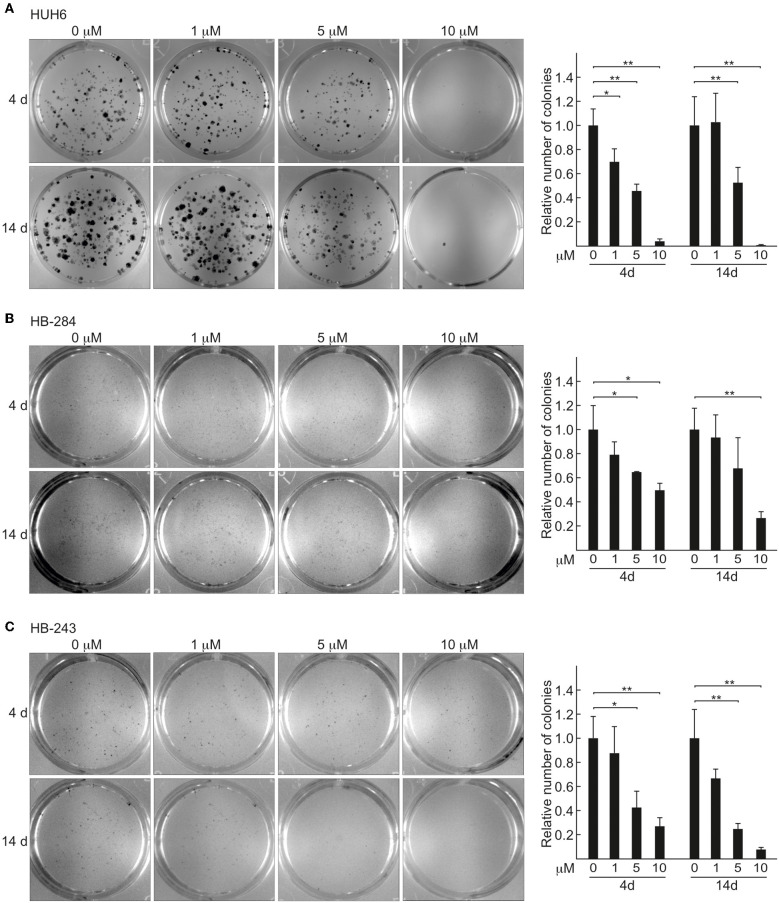
HB 2D cell cultures show decrease in viability and clonogenic potential after CQ exposure. Relative number of colonies after 4 and 14 days of CQ treatment: HUH6 **(A)**, HB-284 **(B)**, and HB-243 **(C)**. Colonies stained with crystal violet after 4 or 14 days CQ administration. CQ; 1, 5, and 10 μM. *P < 0.05, **P < 0.01. One-way ANOVA was utilized to measure statistical significance. Bar plots are presented as relative values of mean ± RSD (*N* = 3).

Due to low cell density, spheroids are not suitable for many high throughput applications. To circumvent this limitation, we validated our 3D spheroid findings in HB cells cultured in a 2D format. Clonogenic potential was measured after short term (4 days) and long term (14 days) CQ administration. A statistically highly significant decrease in clone formation was evident already after 4 days of treatment at concentrations of 5 and 10 μM (Figures 2A–C), and long-term exposure had the same effect on cell viability (Figures 2A–C).

### CQ Treatment Depletes NAD^+^ and Aspartate in HUH6 Cells

**Figure 3:**
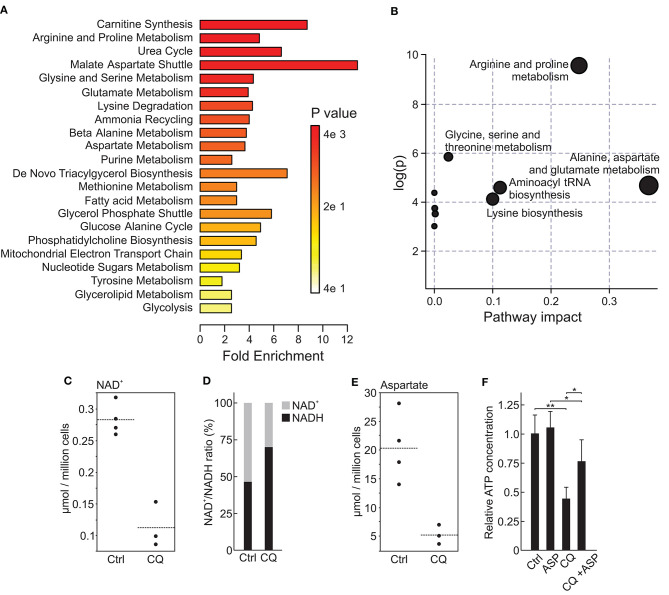
Metabolomic alterations in HUH6 cells treated with 5 μM CQ. Enrichment analysis showing the most abundantly altered metabolite sets **(A)**. Pathway impact analysis demonstrating the importance of altered metabolites for pathway functionality **(B)**. NAD^+^ concentrations **(C)** and NAD^+^/NADH **(D)** ratio in CQ treated HUH6 cells compared to untreated control cells. Aspartate concentrations **(E)** in CQ treated cells compared to untreated control cells. Impact of aspartate supplementation on CQ treated or untreated HUH6 cell viability **(F)**. Single metabolites values are given as normalized concentration (μmol/million cells). NAD^+^/NADH ratio is presented as percentages of normalized total NAD concentrations. Bar plots are presented as relative values of mean ± RSD, (*N* = 3). ASP; 10 mM aspartate. Metabolite analyses were performed with MetaboAnalyst 4.0. *P < 0.05, **P < 0.01.

Inhibition of autophagic flux is one of the known mechanisms of CQ action, and dysfunction in this process leads to a shortage of nutrients in cancer cells (53). Metabolomic profiling of HUH6 cell line treated with 5 μM CQ revealed a statistically significant decrease in 12 metabolites and an increase in 4 metabolites (Table 2). Metabolite enrichment set analysis denoted metabolites associated with malate-aspartate shuttle to be the most prominently altered (Figure 3A). Pathway analysis implicated alanine, aspartate, and glutamate metabolism as having the highest impact (Figure 3B). At the single metabolite level, nicotine adenine dinucleotide (NAD^+^) was the most significantly altered with a 60% decrease in concentration after CQ treatment (Figure 3C). In further investigation, NAD^+^/NADH ratio was noted to be 2.9-fold higher (*p* < 0.02) in untreated cells compared to CQ treated cells (Figure 3D). Aspartate, which was highlighted both in metabolite enrichment and pathway analyses, demonstrated 75% decrease in concentration when comparing CQ treated cells to control cells (Figure 3E). Closer evaluation demonstrated that aspartate supplementation was able to prevent cell death triggered by CQ treatment. Cell viability was 1.7-fold higher when cells were treated with 5 μM of CQ supplemented with aspartate compared to CQ treatment (Figure 3F). Aspartate supplementation alone had no effect on cell viability (Figure 3F). Normalized metabolite concentrations are shown in Supplementary Table 2.

**Table 2:** The most altered metabolites after 96 h CQ (5 μM) exposure.

**Metabolite**	**FC**	**Log_**2**_(FC)**	***P*-value**
NAD^+^	0.39903	−1.3254	0.0006958
Myoinositol	0.46644	−1.1003	0.0028352
4-Pyridoxic acid	3.1111	1.6374	0.0049283
Lysine	3.043	1.6055	0.0075209
Aspartate	0.25574	−1.9672	0.0087533
Betaine	0.095446	−3.3892	0.0096113
Arginine	2.5807	1.3677	0.011984
GABA	0.10494	−3.2524	0.027384
Creatine	0.32802	−1.6081	0.027633
Isovalerylcarnitine	0.274	−1.8678	0.027858
ADMA	2.5424	1.3462	0.028598
Carnitine	0.14461	−2.7898	0.029634
Phosphoethanolamine	0.12009	−3.0578	0.032194
Hydroxyproline	0.41124	−1.282	0.040055
Taurine	0.37727	−1.4063	0.04041
cAMP	0.45375	−1.14	0.041231
Cystathionine	0.34233	−1.5465	0.050692
Octanoylcarnitine	0.4	−1.3219	0.052182
Decanoylcarnitine	0.27586	−1.858	0.064599
Hexanoylcarnitine	0.39548	−1.3383	0.066445

### Death-Associated Gene Expression Changes in HUH6 Cells Exposed to CQ

**Figure 4:**
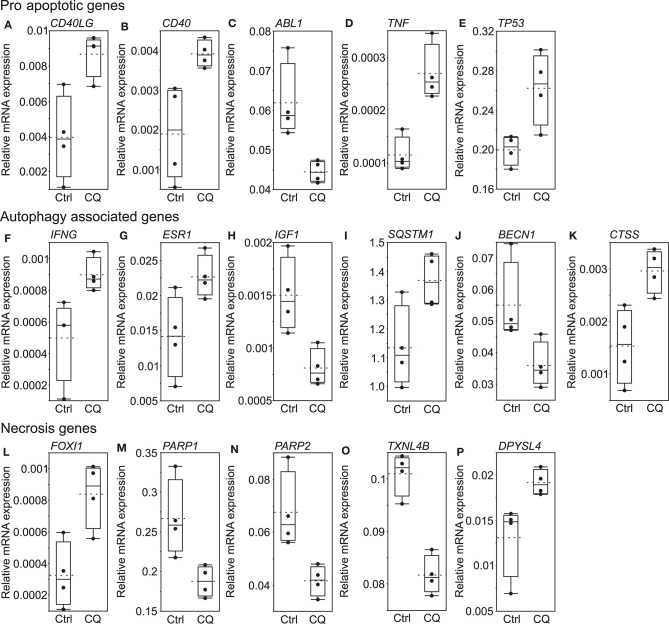
Significantly altered genes detected by Cell Death Pathway Finder array. Pro-apoptotic genes **(A–E)**, autophagy associated genes **(F–K)**, necrosis genes **(L–P)**. Black dots represent normalized expression of independent samples, the box represents the interquartile range, and the whiskers represent the first and fourth quartile. The solid line is median and dashed line shows mean expression of each gene after 96 h of 5 μM CQ or control treatment (*N* = 4).

RT^2^ Profiler Cell Death Pathway Finder array recognized 16 out 84 genes (Figure 4) to be statistically significantly differentially expressed in HUH6 cells treated with 5 μM CQ for 96 h. Six of these genes were classified by the manufacturer as pro-apoptotic genes (Figures 4A–F: *CD40LG, CD40, TNF, TP53, IFNG*, and *ABL1)*, five as autophagy related (Figures 4G–K: *ESR1, IGF1, SQSTM1, BECN1*, and *CTSS*), and five as necroptotic (Figures 4L–P: *PARP1, PARP2, FOXI1, TXNL4B*, and *DPYSL4*). Normalized gene expression of the whole array panel is shown in Supplementary Table 2.

### CQ Inhibits PARPs in HB Cells

**Figure 5:**
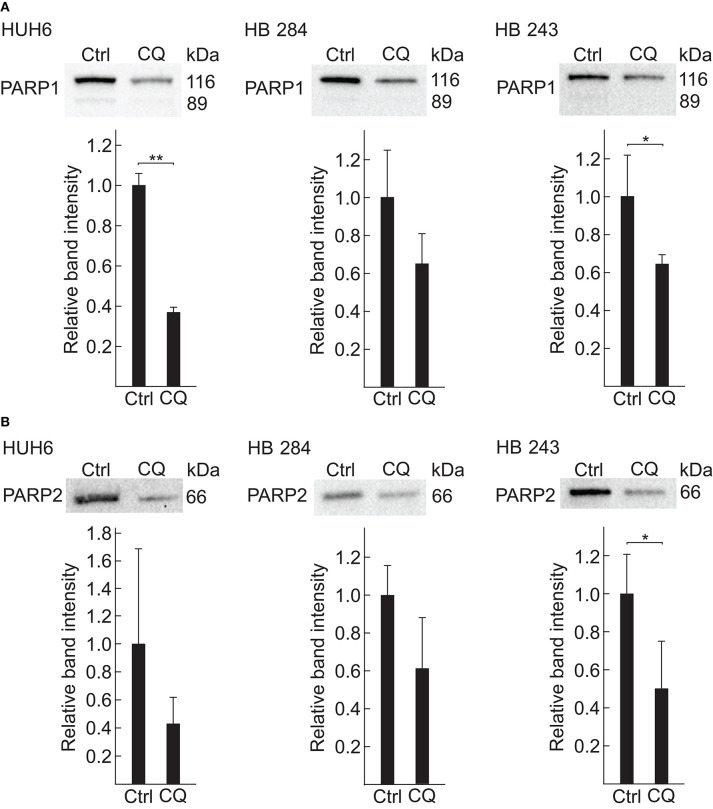
PARP1 and PARP2 are downregulated after 96 h CQ exposure in HB cells. Relative PARP1 **(A)** and PARP2 **(B)** protein expression in HUH6 (left panel), HB-284 (middle panel), and HB-243 (right panel) cells treated with 5 μM CQ detected with western blotting. Normalized band intensity of three independent sample in each group were used to calculate relative protein expression. Student's *t*-test was exploited for statistical analysis. *P < 0.05, **P < 0.01. Bar plots are presented as relative values of mean ± RSD.

Given that the most significant alteration found by metabolomic profiling was a drastic decrease in NAD^+^ concentration and that poly(ADP)-ribose polymerase (PARP) function is highly NAD^+^-dependent (54), we chose *PARP1* and *PARP2* (Figures 4M,N) for further analyses. HUH6, HB-284, and HB-243 cells were treated with 5 μM of CQ for 96 h and subsequently quantitative western blotting analysis was performed. Statistically significant decrease in PARP1 protein expression was detected in HUH6 and HB-243 cell lines (Figure 5A), over 60 and 30% reduction, respectively. Furthermore, immunofluorescence staining revealed decreased nuclear expression of PARP1 after CQ treatment compared to control cells (Supplementary Figures 2D–F). Similarly, PARP2 expression was observed to be markedly decreased in cells treated with CQ (Figure 5B).

## Discussion

Chemotherapy resistance is the major obstacle limiting HB patient survival (10). Therefore, novel treatment strategies are needed for patients with advanced and chemoresistant HB. We found robust antineoplastic activity of CQ monotherapy in HB tumor spheroid models. Further, our results shed light on the molecular mechanisms of the CQ. To this end, our findings describe the CQ administration associated gene expression alterations in cell death related pathways, especially the inhibition of PARP1 and PARP2, as well as metabolomic perturbations leading to failure in NAD^+^ balance and aspartate availability.

Spheroids mimic 3D architecture of solid tumors and offer tissue-like environment for cancer drug research (55). The patient-derived HB models used here recapitulate the characteristics of advanced and aggressive original HB tumors (50). Taken together, our results demonstrate decreased cell viability and activation of apoptotic cell death as response to CQ treatment in these HB cell models. Although spheroids have multiple advantages compared to the traditional 2D cell cultures, they still lack responses produced by multicellular organ system. Organoids may better represent the complexity of tumor and neighboring tissues (56), and in the future, these findings should be confirmed in HB organoids.

Aspartate depletion induced by CQ treatment was recently demonstrated in pancreatic cancer cells restricting nucleotide biosynthesis and subsequently predisposing cells to the replication stress (57). Additionally, in oxygen deprived environment, typical for solid tumors, aspartate has been suggested to be a limiting metabolite for proliferation (58, 59). In line with these previous observations, the present findings demonstrate reduced aspartate availability and simultaneous decrease in cell viability of HB cells treated with CQ. Moreover, aspartate supplementation rescued cells from CQ induced cell death suggesting that aspartate availability may be one the crucial targets of this treatment.

Aspartate biosynthesis requires electron acceptors, e.g., NAD^+^, and in the presence of oxygen their pools are maintained by electron transport chain (ETC) reactions (58, 59). ETC is carried out by four complexes (I–IV) and ATP synthase. Complex I regenerates NAD^+^ from NADH and pharmacological inhibition of that reaction is linked with disturbed NAD^+^/NADH balance and subsequent reduction in aspartate synthesis (59). We noted a contemporaneous drop in aspartate and NAD^+^ concentrations in HB cells treated with CQ, implying that limited aspartate availability may be a consequence of ETC malfunction. In addition, NAD^+^/NADH balance shifted toward NADH following CQ treatment, suggesting that CQ impacts NAD oxidation. Further investigations are needed to determine whether inhibition of mitochondrial complex I is a bona fide mechanism of CQ action in HB cells.

PARPs are multifunctional enzymes involved in epigenetic modifications, signal transduction, stress sensing, and DNA repair (60–62). Interestingly, in a context of aggressive HB, PARP1 was recently shown to be aberrantly activated, promoting expression of nonfunctional tumor suppressor proteins (63). Herein, we demonstrated decreases in PARP1 and PARP2 expression in HB cells both at mRNA and protein level after CQ treatment. Since the catalytic activities of PARP1 and PARP2 are NAD^+^ dependent (54), we suggest that reduced NAD^+^ pools trigger degradation of PARPs. Moreover, CQ may downregulate aberrantly expressed tumor suppressor proteins by PARP inhibition compromising HB cell survival. The PARP inhibitor (PARPi) talazoparib has been shown to synergize with CQ in a pediatric chronic myeloid leukemia mouse model (64). Another PARPi, niraparib, combined with CQ exhibited increased cytotoxicity in hepatocellular carcinoma *in vitro* and *in vivo* (65). It would be of interest to study whether a similar synergism exists also in HB models.

**Figure 6:**
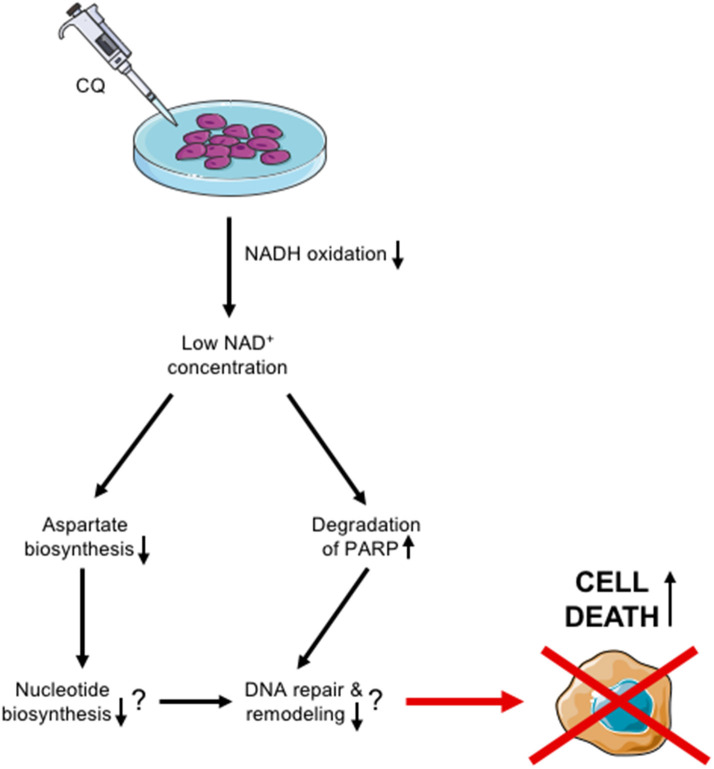
Schematic view of proposed CQ action in HUH6 cells.

All in all, our results suggest that CQ has potential as an additional treatment modality for aggressive HB through the mechanisms summarized in Figure 6. Thus, this pre-clinical study sets the basis for further investigations in HB and offers novel potential applications for CQ re-purposing strategies.

## Conclusions

CQ treatment in relevant concentrations decreases viability of HB spheroids. Further, the spheroids established from chemoresistant tumors exhibited increased apoptotic activity after CQ treatment, suggesting that CQ holds potential in treatment of aggressive HB. Moreover, our study demonstrates disturbance in NAD^+^ and aspartate metabolism exposing cells to impaired DNA repair and histone remodeling by PARPs.

## Data Availability Statement

All datasets presented in this study are included in the article/Supplementary Material.

## Author Contributions

KE, MH, and MP: conceptualization. KE, SC, DW, and MP: methodology. KE, EL, TS, AK, and MP: acquisition, analysis, or interpretation of data. MH: funding acquisition. SC and J-GJ: resources. DW, MH, and MP: supervision. KE: writing-first draft. SC, EL, TS, AK, J-GJ, DW, MH, and MP: writing-review and editing. KE, SC, EL, TS, AK, J-GJ, DW, MH, and MP: final approval of the manuscript version to be published. All authors contributed to the article and approved the submitted version.

## Conflict of Interest

SC is employed by the company XenTech and J-GJ is the president of the company XenTech. The remaining authors declare that the research was conducted in the absence of any commercial or financial relationships that could be construed as a potential conflict of interest.

## Abbreviations

ABL1, ABL Proto-Oncogene 1: Non-Receptor Tyrosine Kinase
ASP: Aspartate
B2M: Beta-2-Microglobulin
BECN1: Beclin-1
CD40: CD40 Molecule
CD40LG: CD40 Ligand
CQ: Chloroquine
CTSS: Cathepsin S
DPYSL4: Dihydropyrimidinase Like 4
ESR1: Estrogen Receptor 1
ETC: Electron transport chain
FAP: Familial adenomatous polyposis
FOXI1: Forkhead Box I1
GAPDH: Glyceraldehyde-3-Phosphate Dehydrogenase
HB: Hepatoblastoma
HPRT1: Hypoxanthine Phosphoribosyltransferase 1
IFNG: Interferon gamma
IGF1: Insulin Like Growth Factor 1
NAD^+^: Nicotinamide adenine dinucleotide
NADH: Nicotinamide adenine dinucleotide hydrogen
PARP1: Poly(ADP)-ribose polymerase 1
PARP2: Poly(ADP)-ribose polymerase 2
PARPi: Poly(ADP)-ribose polymerase inhibitor
SQSTM1: Sequestosome 1
TNF: Tumor necrosis factor
TP53: Tumor Protein P53
TXNL4B: Thioredoxin Like 4B.
