# Supplementary material for: Chloroquine Triggers Cell Death and Inhibits PARPs in Cell Models of Aggressive Hepatoblastoma
Source: Front Oncol. 2020 Jul 17;10:1138. doi: 10.3389/fonc.2020.01138 (PMC7379510; doi:10.3389/fonc.2020.01138)
Supplement: Supplementary file 1 [file Table_1.pdf]

Supplementary table 1. Clinical trials of CQ administration in oncology (clinicaltrials.gov, visited 8.4.2020).

NCT number	Title	Status	Study Results	Conditions	Interventions
NCT01575782	Chloroquine as an Anti-autophagic Radiosensitizing Drug in Stage I-III Small Cell Lung Cancer	Terminated	No Results Available	Small Cell Lung Cancer	Drug: Chloroquine
NCT02333890	A Phase 2 Randomized, Double-blind Trial Evaluating the Effects of Chloroquine in Breast Cancer	Unknown status	No Results Available	Breast Cancer, Invasive Breast Cancer	Drugs: Chloroquine, Placebo
NCT00969306	Chloroquine as an Anti-Autophagy Drug in Stage IV Small Cell Lung Cancer (SCLC) Patients	Terminated	No Results Available	Small Cell Lung Cancer	Drug: Chloroquine, A-CQ 100
NCT01446016	Chloroquine With Taxane Chemotherapy for Advanced or Metastatic Breast Cancer After Anthracycline Failure (CAT)	Completed	No Results Available	Breast Neoplasms, Breast Cancer	Drugs: Taxane, Docetaxel, Abraxane, Ixabepilone
NCT02071537	Chloroquine in Combination With Carboplatin/Gemcitabine in Advanced Solid Tumors	Completed	No Results Available	Malignant Neoplasm, Solid Tumors	Drugs: Chloroquine, Carboplatin, Gemcitabine
NCT01777477	Adjuvant Effect of Chloroquine on Gemcitabine	Completed	No Results Available	Pancreatic Cancer	Drugs: Chloroquine, Gemcitabine
NCT04333914	Prospective Study in Patients With Advanced or Metastatic Cancer and SARS-CoV-2 (COVID-19) Infection	Recruiting	No Results Available	SARS-CoV-2 (COVID-19) Infection|Advanced or Metastatic Hematological or Solid Tumor	Drugs: Chloroquine analog (GNS651), Nivolumab, Tocilizumab; Other: Standard of care
NCT01023477	Study of the Efficacy of Chloroquine in the Treatment of Ductal Carcinoma in Situ (The PINC Trial)	Completed	No Results Available	Carcinoma, Intraductal, Noninfiltrating Ductal Carcinoma In Situ	Drugs: Chloroquine Standard Dose (500mg/week), Chloroquine Low Dose (250mg/week, Procedure: Breast Biopsy
NCT02496741	Metformin And Chloroquine in IDH1/2-mutated Solid Tumors	Completed	No Results Available	Glioma, Cholangiocarcinoma, Chondrosarcoma	Drugs: Metformin and chloroquine combination
NCT02786589	Plasmodium Immunotherapy for Lung Cancer	Recruiting	No Results Available	Lung Cancer, Non-small Cell	Biological: Blood-stage infection of P. vivax
NCT01894633	Study of Whole-brain Irradiation With Chloroquine for Brain Metastases	Terminated	No Results Available	Brain Metastasis	Drug: Chloroquine; Radiation: Total brain radiotherapy
NCT01727531	IDO2 Genetic Status Informs the Neoadjuvant Efficacy of Chloroquine (CQ) in Brain Metastasis Radiotherapy	Completed	No Results Available	Brain Metastasis	Drug: Chloroquine diphosphate

NCT01438177	Chloroquine in Combination With VELCADE and Cyclophosphamide for Relapsed and Refractory Multiple Myeloma	Terminated	Has Results	Multiple Myeloma	Drug: Velcade, Cyclophosphamide, Chloroquine
NCT01469455	DNA Repair Inhibitor & Irradiation on Melanoma	Completed	No Results Available	Local Metastatic Melanoma	Drug: DT01
NCT03243461	International Cooperative Phase III Trial of the HIT-HGG Study Group (HIT-HGG-2013)	Recruiting	No Results Available	Glioblastoma WHO Grade IV, Diffuse Midline Glioma Histone 3 K27M WHO Grade IV, Anaplastic Astrocytoma WHO Grade III, Diffuse Intrinsic Pontine Glioma, Gliomatosis Cerebri	Drugs: Temozolomide + Valproic Acid, Temozolomide + Chloroquine
NCT02366884	Clinical Evaluation of a New Form of Cancer Therapy Based on the Principles of Atavistic Metamorphosis (Atavistic Chemotherapy).	Recruiting	No Results Available	Neoplasms	Drugs: Anti-Bacterial Agents, Anti-Fungal Agents, Anti-Protozoal Agents
NCT02378532	The Addition of Chloroquine to Chemoradiation for Glioblastoma	Completed	No Results Available	Glioblastoma Multiforme	Drugs: Chloroquine, Temozolomide; Radiation: Radiotherapy
NCT02432417	The Addition of Chloroquine to Chemoradiation for Glioblastoma	Not yet recruiting	No Results Available	Glioblastoma, Astrocytoma, Grade IV	Drug: Chloroquine
NCT00224978	Chloroquine for Treatment of Glioblastoma Multiforme	Completed	No Results Available	Glioblastoma Multiforme	Drug: Chloroquine
NCT03400865	Cabergoline Combined Hydroxychloroquine/Chloroquine to Treat Resistant Prolactinomas	Not yet recruiting	No Results Available	Resistance, Disease; Prolactinoma	Drugs: HCQ/CQ and CAB combined treatment
NCT03979651	MEK and Autophagy Inhibition in Metastatic/Locally Advanced, Unresectable Neuroblastoma RAS (NRAS) Melanoma	Not yet recruiting	No Results Available	Metastatic NRAS Melanoma	Drugs: Trametinib plus hydroxychloroquine in patients with NRAS Melanoma (Dose 1), Trametinib plus hydroxychloroquine in patients with NRAS Melanoma (Dose 2), Trametinib plus hydroxychloroquine in patients with NRAS Melanoma (Dose 3)
