# Supplementary material for: Chloroquine Triggers Cell Death and Inhibits PARPs in Cell Models of Aggressive Hepatoblastoma
Source: Front Oncol. 2020 Jul 17;10:1138. doi: 10.3389/fonc.2020.01138 (PMC7379510; doi:10.3389/fonc.2020.01138)
Supplement: Supplementary file 3 [file Image_1.pdf]

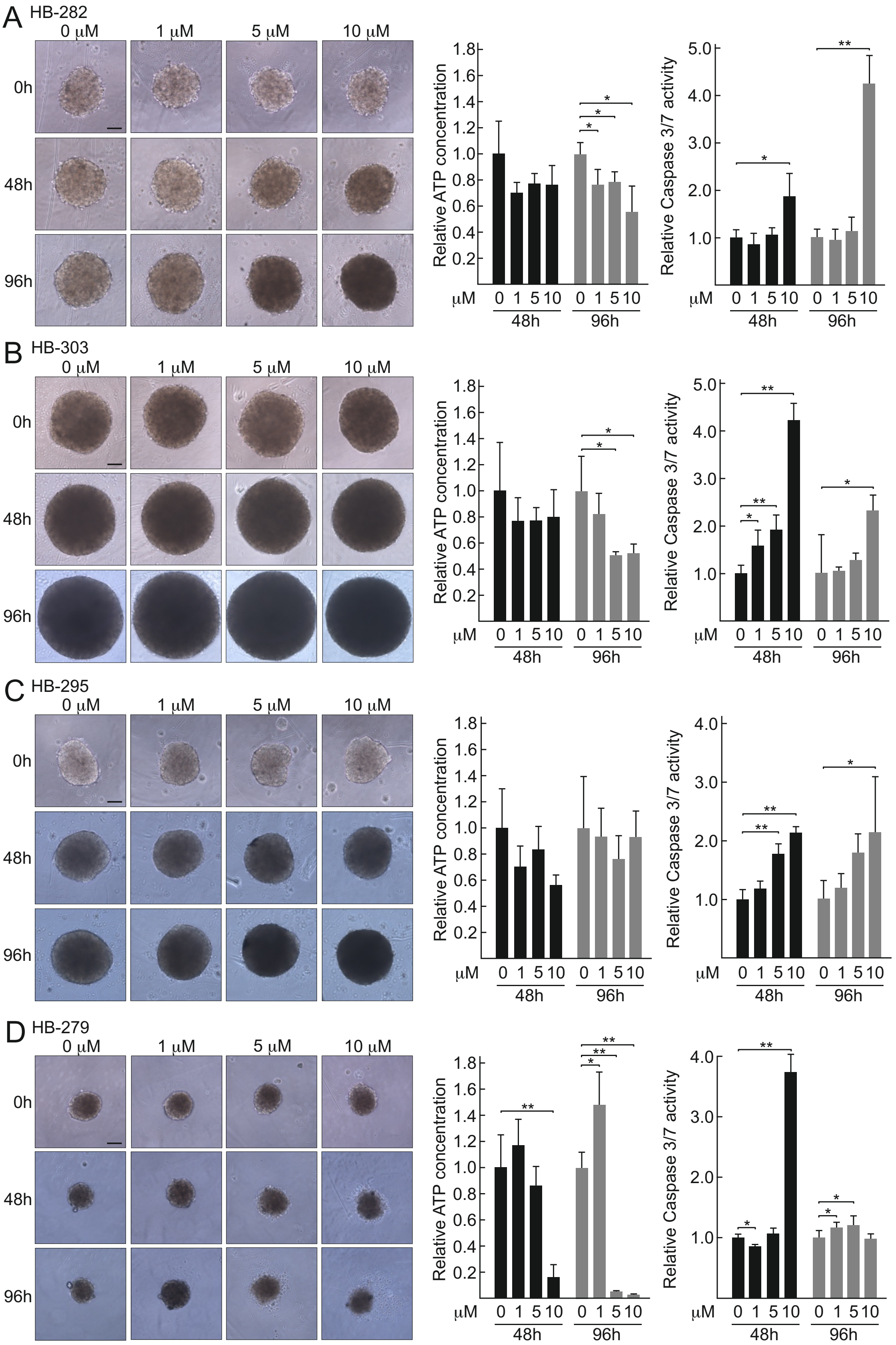

Supplementary figure 1. Morphology, viability, and caspase 3/7 activation of HB spheroids treated with CQ. Morphology, relative ATP concentration, and caspase activation of HB-282 **(A)**, HB-303 **(B)**, HB-295 **(C)**, and HB-279 **(D)** derived spheroids treated with control medium (0 μM) or CQ at concentrations of 1 μM, 5 μM, and 10 μM. CQ concentrations; 1 μM, 5 μM, and 10 μM. *P < 0.05. **p < 0.01. Bar plots are presented as relative values of mean ± RSD (N = 3). Pictures were captured at initiation of treatment (0 h) and after 48 h and 96 h of CQ administration. Magnification 10x, scale bar = 10 μm.
