# Supplementary material for: Chloroquine Triggers Cell Death and Inhibits PARPs in Cell Models of Aggressive Hepatoblastoma
Source: Front Oncol. 2020 Jul 17;10:1138. doi: 10.3389/fonc.2020.01138 (PMC7379510; doi:10.3389/fonc.2020.01138)
Supplement: Supplementary file 4 [file Image_2.pdf]

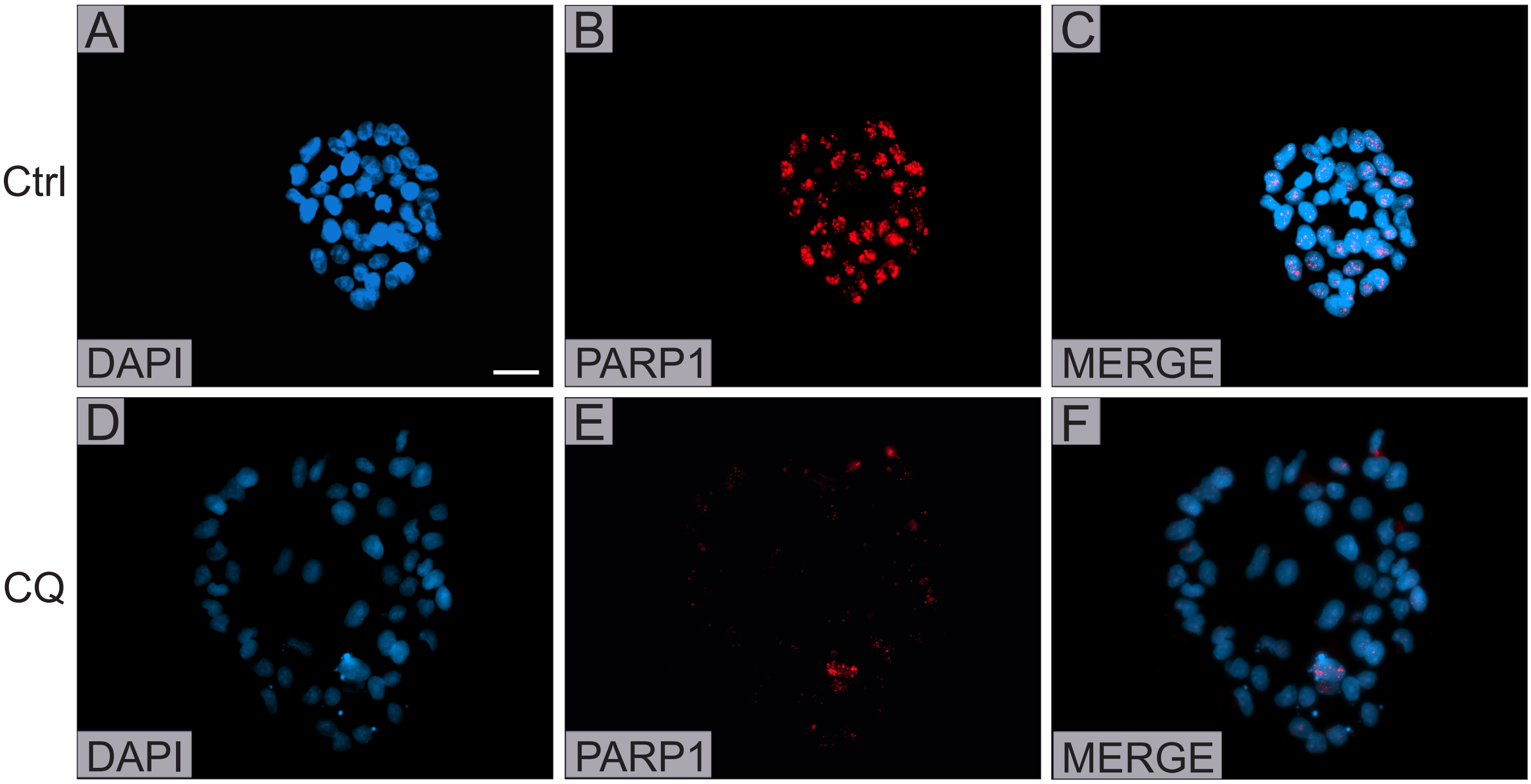

Supplementary figure 2. CQ decreases nuclear PARP1 expression. Immunofluorescence staining of PARP1 demonstrates decreased nuclear protein expression after 96 h CQ (5 μM) treatment (D-F) compared to controls (A-C) in HUH6 cells. Scale bar = 20 μm.
